# Modified Fisher method for unilateral cleft lip-report of cases

**DOI:** 10.1186/s40902-017-0109-1

**Published:** 2017-05-05

**Authors:** Hui Young Kim, Joonhyoung Park, Ming-Chih Chang, In Seok Song, Byoung Moo Seo

**Affiliations:** 1 Department of Oral and Maxillofacial Surgery, Dental Research Institute, School of Dentistry, Seoul National University, 101 Daehakro, Jongro-Gu, Seoul, 03080 Korea; 2 Sijhih Cathay General Hospital, New Taipei City, Taiwan; 3 Department of Oral and Maxillofacial Surgery, Anam Hospital, Korea University, Seoul, Korea

**Keywords:** Cleft lip, Fisher, Orbicularis oris muscle, Primary rhinoplasty, Alar web correction

## Abstract

**Background:**

Rehabilitation of normal function and form is essential in cleft lip repair. In 2005, Dr. David M. Fisher introduced an innovative method, named “an anatomical subunit approximation technique” in unilateral cleft lip repair. According to this method, circumferential incision along the columella on cleft side of the medial flap is continued to the planned top of the Cupid’s bow in straight manner, which runs parallel to the unaffected philtral ridge. Usually, small inlet incision is needed to lengthen the medial flap. On lateral flap, small triangle just above the cutaneous roll is used to prevent unesthetic shortening of upper lip. This allows better continuity of the Cupid’s bow and ideal distribution of tension.

**Case presentation:**

As a modification to original method, orbicularis oris muscle overlapping suture is applied to make the elevated philtral ridge. Concomitant primary rhinoplasty also results in good esthetic outcome with symmetric nostrils and correction of alar web. As satisfactory results were obtained in three incomplete and one complete unilateral cleft lip patients, indicating Fisher’s method can be useful in cleft lip surgery with functional and esthetic outcome.

**Conclusions:**

Clinically applied Fisher’s method in unilateral cleft lip patients proved the effectiveness in improving the esthetic results with good symmetry. This method also applied with primary rhinoplasty.

## Background

Rehabilitation of normal function and form is essential in cleft lip repair. To attain this goal, various methods have been developed and advocated. Among them, rotation advancement technique, so called Millard method, has been widely used because of lengthening effect and preserving more adequate Cupid’s bow dimple component than previous triangular flaps [1]. However, flap design curving under the columella base might create unfavorable scar line across the philtral ridge especially in some people who have tendency to make hypertrophy and keloid.

**Fig. 1:**
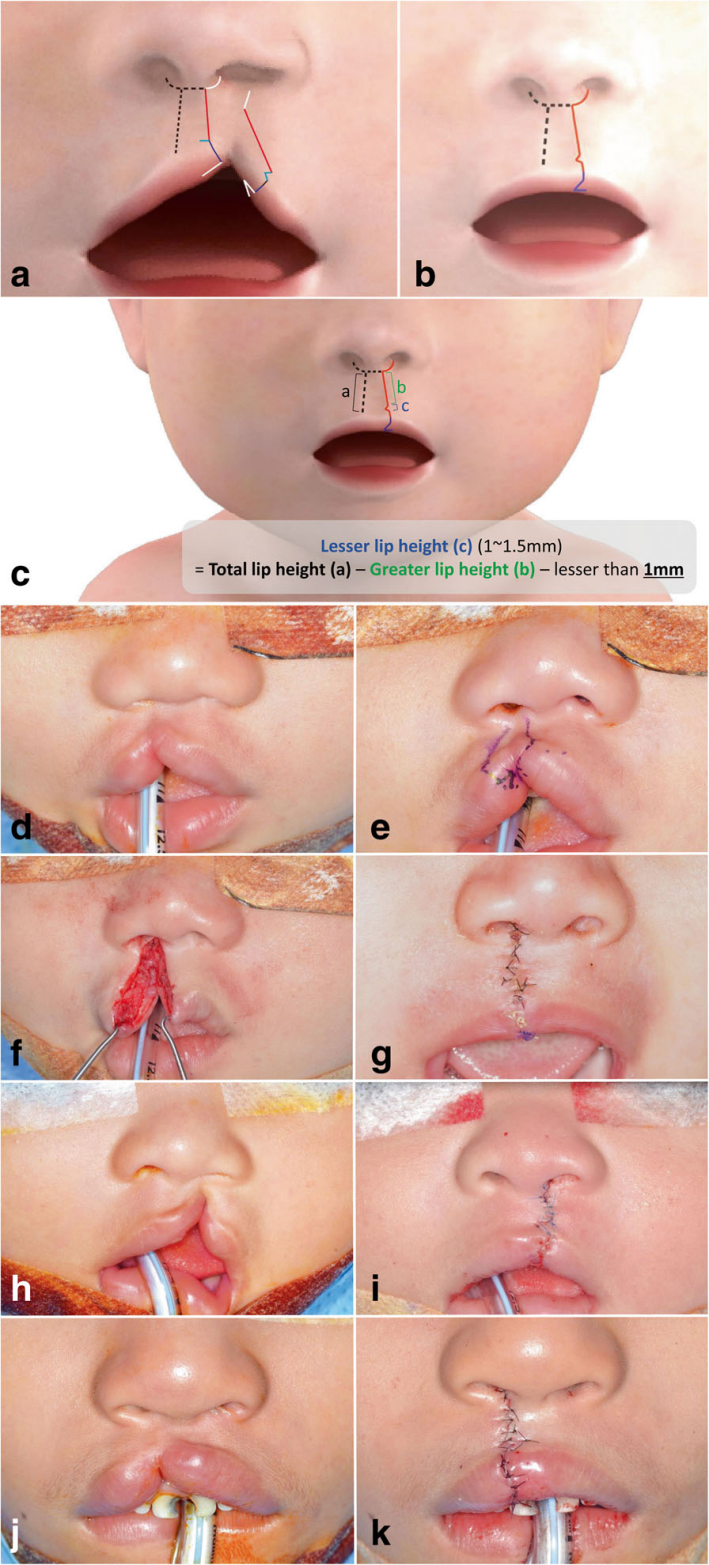
Incomplete unilateral cleft lip. **a** Surgical design: *dotted line* represents noncleft side normal anatomical landmarks. Circumferential incision along the columella and philtral ridge should be symmetric to the normal side. Each length of incision lines with same color is equal. Inlet incision on medial flap of cleft margin (*blue line*) has same length with each side of small triangle. This small triangle should be just above the cutaneous roll, and if there is enough length in lateral flap of cleft side, then small triangle flap might be omitted. **b** After approximation, scar line on the philtral ridge of cleft side is less visible than other methods. **c** Cleft side lateral lip element can be lengthened by small triangular flap and Rose-Thompson effect. Therefore, the length of the lesser lip height, which means the base of the small triangle, should be 1 mm less than the greater lip height. **d-g** Nine-month-old boy with incomplete unilateral cleft lip (case 1). **h**, **i** Six-month-old boy with incomplete unilateral cleft lip (case 2). **j**, **k** Five-year-old boy with incomplete unilateral cleft lip (case 3)

In 2005, Dr. David M. Fisher [2] introduced an innovative method, named “an anatomical subunit approximation technique” in unilateral cleft lip repair (Fig. 1 a–c). According to this method, circumferential incision along the columella on cleft side of the medial flap is continued to the planned top of the Cupid’s bow in straight manner, which runs parallel to the unaffected philtral ridge. This incision line is made on the cleft side coinciding with the non-cleft side, and the simulated philtral ridge of the cleft side. The proposed peak of the Cupid’s bow on the lateral lip element is Noordhoff’s point on vermilion-cutaneous junction where the cutaneous roll and the vermilion-mucosal junction lines start to converge medially [3]. Usually, small inlet incision is needed to lengthen the cleft side of medial flap. On lateral flap, small triangle (smaller than traditional triangle technique) just above the cutaneous roll is used to prevent unesthetic shortening of upper lip on medial flap. This allows better continuity of the Cupid’s bow and ideal distribution of tension on the lip. In addition to this, muscle overlapping suture is applied to make the elevated philtral ridge.

Fisher’s method has some advantages. First of all, it provides very natural and esthetic columella and philtral ridge due to its symmetric design and lack of incision line across the philtrum. Secondly, cleft side lateral lip element can be lengthened by small triangular flap and Rose-Thompson effect which means slightly concave excision of the cleft margins providing additional length when closing in a straight line (Fig. 1 c). Furthermore, this small triangular flap has not only scar breaking effect but also accentuation effect on the pout of the lip by providing small amount of tension. In our cases, subtle modification of Fisher’s method was introduced to improve lip contour on the philtral ridge; orbicularis oris muscle overlapping. Through this commonly applied modification reconstructed philtral ridge can be augmented to mimic the elevated normal side of the ridge.

## Case presentation

In this article, three incomplete and one complete unilateral cleft lip patients are presented. This study was approved by the Ethics Committee of Seoul National University Graduate School of Dentistry (IRB no. S-D20150022).

### Incomplete unilateral cleft lip

The case 1 was a 9-month-old boy with incomplete unilateral cleft lip on his right side (Fig. 1 d–g). Although the length of tissue supposed to form philtral ridge on affected side is deficient, the difference of lip lengths between affected side and non-affected side was not huge. The lateral and medial side tissue were enough on affected side, and nasal deformity was relatively small. The case 2 was a 6-month-old boy with incomplete unilateral cleft lip on his left side (Fig. 1 h, i). His prolabium and nasal deformities were greater than case 1. He had asymmetric nares, deviated collumella, and cleft side subnasale was located in lower position than normal side. The discontinuity of orbicularis oris muscle cause bulging of prolabium area. Case 3 was a 5-year-old boy with incomplete unilateral cleft lip which was limited on his vermilion area (Fig. 1 j, k). His nasal deformity was not severe, and tissue seems to be enough. The difference of length between medial and lateral lip was slight. His philtrum was also located in the middle of face.

#### Surgical procedure

All the procedures are up to the patient’s condition, but basically it was like below;

Surgical field preparation, routine double head draping was done, and presurgical photographs were taken. The key landmarks and surgical incision line was designed with marking stick and gentian violet dye solution. Local anesthetics were infiltrated on labium and nasal area with 2% lidocaines with 1:100,000 epinephrine. Injection of marked landmarks could make tattooing effect, to facilitate preserving landmarks effectively during surgical procedure. The incision on the medial segment was carried on with a no. 15 blade and redundant cleft marginal tissue was discarded. The skin on the medial segment was undermined from the skin and mucosa to separate orbicularis oris muscle for about 1 mm distal from cut edge. The incision on the lateral segment was carried on with a no. 15 blade, and excess marginal tissue was discarded. Dissection between skin and muscle on lateral element was done as same manner as medial side. The mucosal incision was closed with 5-0 Vicryl®. Upturned orbicularis oris muscle at the alar base of cleft side was approximated to anterior nasal spine with 4-0 PDS®. Orbicularis oris muscle was overlapped and sutured with 5-0 Ethilon®. Medial and lateral lip flaps were approximated at the junction of red vermilion and cutaneous roll and then submucosal closure was done with 5-0 Vicryl®. Skin was closed with 6-0 Ethilon®. Upper vermilion flap was rotated and was sutured with 6-0 Vicryl®. Lip was closed with 5-0 Vicryl®. Infraorbital nerve block anesthesia was done with 2% lidocaine with 1:100,000 epinephrine to reduce postoperative pain. Dressing was done using antibiotic ointment and Steri-strip®.

### Complete unilateral cleft lip

**Fig. 2:**
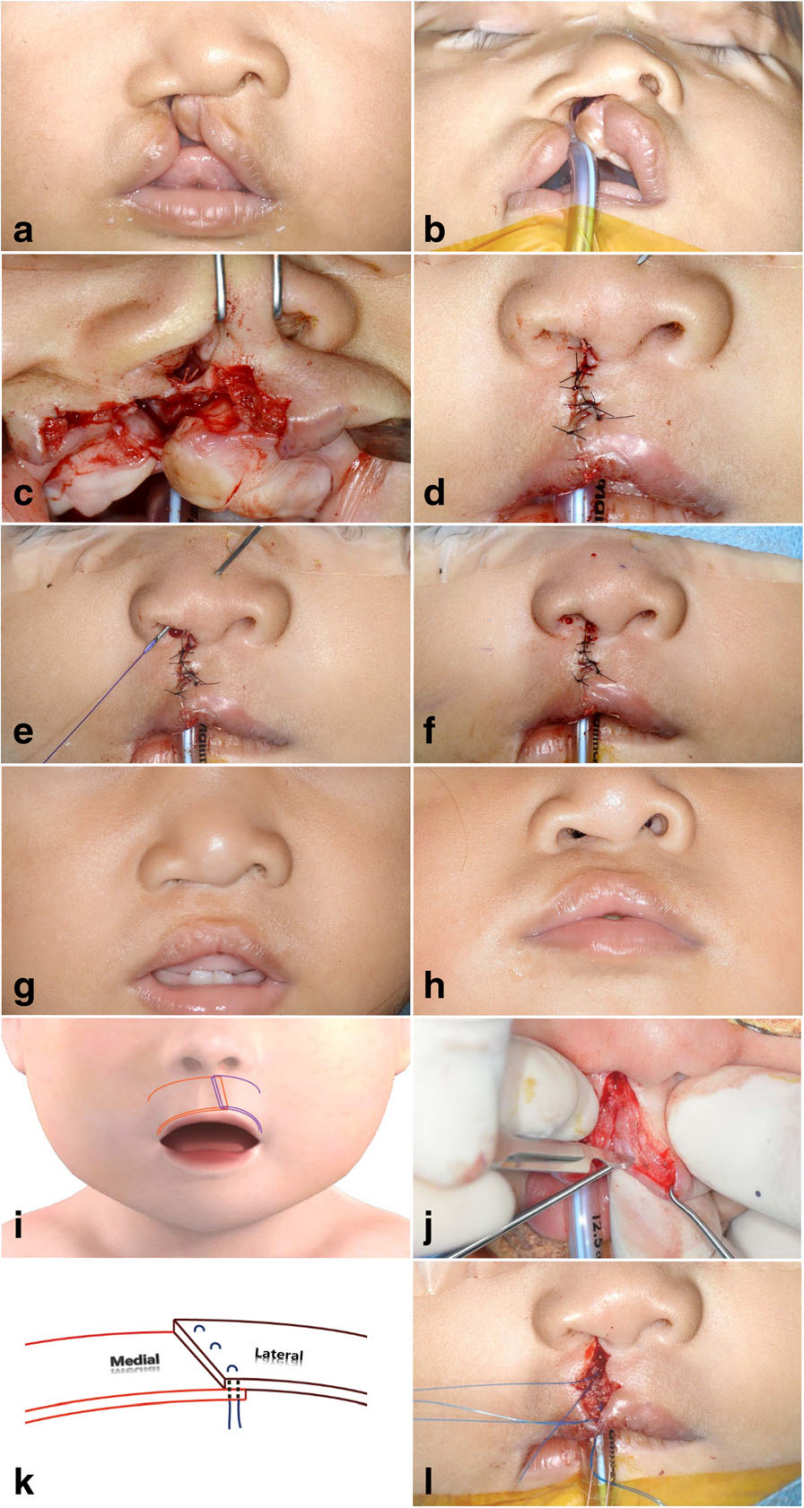
Three-month-old girl with complete unilateral cleft lip (case 4). **a**, **b** Preoperative photos showed complete cleft lip with wide gap and nose deformity. **c** An inferior turbinate releasing incision, medial flap, and modified lateral flap are used to close the nasal side. **d** After lip approximation, symmetric lip height was attained. **e** Alar web correction was performed as a part of primary rhinoplasty with minimally invasive procedure. **f** Post-operative photos showed symmetric nostril with adequate lip height. **g**, **h** Post-operative 1 year photos showed good lip symmetry with natural philtral ridge. Some degree of recurrence of alar web deformity was noticed, which can be corrected after growth completed. **i**, **k** Diagram of orbicularis oris muscle overlapping suture showed that the lateral side was located over medial muscle margin to mimic philtral ridge prominence. **j** Dissection of orbicularis oris muscle from skin and mucosa was performed about 1 mm distal to cut edge. **l** Orbicularis oris muscle was overlapped and sutured with three 5-0 nylon stitches in the manner of deep to superficial and superficial to deep

The case 4 was a 3-month-old girl with complete unilateral cleft lip on her right side (Fig. 2). The principle of skin design is the same with incomplete case. However, in complete cases, more considerations should be addressed to achieve equal circumferential nares and symmetric alar base than in incomplete cases. The circumferential length of noncleft side nare is calculated to determine uppermost point of lateral flap on cleft side. An inferior turbinate releasing incision was done to reduce the buckling effect of lesser segment. In addition, intraoral vestibular incision was made and subperiosteum dissection was carried out superiorly to infraorbital nerve, moreover releasing incision on periosteum was performed to mobilize the lateral flap without tension. The nasal floor was reconstructed by medial, and lateral flap on the base of nose was approximated with 5-0 Vicryl®. The misoriented orbicularis oris muscle toward the columella was dissected and re-orientation was done. Later approximation of medial and lateral flap procedure was similar to the incomplete cases.

### Orbicularis oris muscle overlapping suture

Natural philtral ridge is a key point in rehabilitation of cleft lip patients. The flattening of philtral ridge resulted from failure to reconstruct natural orientation of orbicularis oris muscle during surgery. However, sometimes, even after the approximation of orbicularis oris muscle, we could observe that the outcome of philtral ridge is not prominent enough.

Orbicularis oris muscle overlapping suture can reproduce the natural balanced appearance by making elevated philtral ridge on the cleft side. After dissecting orbicularis oris muscle from skin for about 1 mm distal from the cut edge, lateral segment muscle was overlapped on medial segment of muscle. And then, several 5-0 nylon sutures were made in the manner of deep to superficial and superficial to deep [4] (Fig. 2 i, k).

### Primary rhinoplasty

The abnormal insertion of orbicularis oris muscle into base of columella in cleft lip creates unfavorable forces to pull columella and caudal nasal septum to the non-affected side. Furthermore, insertion of orbicularis oris muscle into the subalar cartilage forces the cleft side of the nose to retract laterally and inferiorly, which results in flattening of the lower lateral alar cartilage. Correction of these anatomic displacement is indispensable to restore normal nasal structure. It is bound up with dissecting, releasing, and repositioning of lower lateral alar cartilage from the margin of medial and lateral flaps on the base of nose. After completion of lip nose skin suture, trans alar cartilage suture initiates from intranasal mucosa and passes lower lateral cartilage and anchors to dermis of nasal skin with 4-0 PDS® [4] (Fig. 2e).

## Discussion

Historically, there are numerous surgical techniques to repair cleft lip from simple cutting to current microscopic rehabilitation. The variety of skin designs were used and were published in cleft lip surgery according to surgeon’s concept. The majority of oral and maxillofacial surgeons in Korea use Millard’s method and its modifications due to advantages in advancement of lateral flap and acceptable outcome. Despite of some modifications, Millard’s method still has weakness because the course of incision line below nostril is almost vertical to the philtral ridge. Although it is positioned just below the nostril side, scar could be visible due to its direction crossing the philtrum [5]. Fisher’s method which was published in 2005 seems to overcome this drawback. It forms exact mirror image of normal side of philtrum to the cleft side philtrum. Some surgeons might doubt about its lengthening effect. They might argue that it is not sufficient to get enough tissue from lateral flap due to limitation of straight method. However, straight method quite overcome its weakness through various modifications, and Fisher’s method also showed its usefulness and esthetic results [6].

Furthermore, adequate approximation of orbicularis oris muscle plays important role in reconstruction of natural philtral ridge which is composed with the philtral groove and the flanking ridges [7]. According to this need, various methods were introduced to repair discontinued orbicularis oris muscle. Cakir et al. [8] reported ‘jack-like eversion by splitting the orbicularis oris muscle’ and Cho [9] reported vertical interdigitation of the orbicularis oris muscle. These methods may not be easily applied to the cleft lip patients because the orbicularis oris muscle is very thin and small especially in 3-month-old babies.

The cleft nasal deformity is not only present at birth, but also proceeds over growth. Most of the surgeons agree that aggressive surgery is not necessary in primary surgical intervention of cleft lip [10]. However, repositioning of cleft side dome and lateral crus of lower lateral cartilage could be performed in minimally invasive manner during primary surgery as we presented here.

In this report of cases, the skin designs were basically followed by Fisher’s proposal, but some modifications were performed. First, acute triangular flap incision line on the vermilion is replaced by broad-based triangular flap to minimize the visible scar line. This wide triangular shaped flap on the vermilion contained some fibers of orbicularis oris muscle to reinforce the vermilion height which can be trimmed and adjusted during approximation procedure. Second, orbicularis oris muscle overlapping suture was applied to make natural looking elevated philtral ridge and to obtain more esthetic outcomes. Finally, we added alar web correction in the case of complete cleft lip to generate symmetrical nostrils.

## Conclusions

Cleft lip repair necessarily follows some principles. Most of all, reconstruction of natural appearance with esthetic scar is mandatory in considering flap design. In addition, functional rehabilitation by muscle reorientation should be obtained through complete understanding of anatomical structures. In these points of view, Fisher’s method presents excellent capability and can be applied for treating unilateral cleft lip patients regardless their extensiveness.
